# Bing-Neel Syndrome, a Rare Presentation of Waldenström Macroglobulinemia—A Multicenter Report by the Polish Lymphoma Research Group

**DOI:** 10.3390/jcm11154447

**Published:** 2022-07-30

**Authors:** Joanna Drozd-Sokołowska, Anna Waszczuk-Gajda, Magdalena Witkowska, Elżbieta Sienkiewicz, Anna Kopińska, Agnieszka Kołkowska-Leśniak, Joanna Barankiewicz, Monika Długosz-Danecka, Piotr Smolewski, Grzegorz Helbig, Ewa Lech-Marańda, Wojciech Jurczak, Przemysław Biecek, Sebastian Giebel, Wiesław Wiktor-Jędrzejczak, Grzegorz Basak

**Affiliations:** 1 Department of Hematology, Transplantation and Internal Medicine, Medical University of Warsaw, Banacha 1a Str., 02-097 Warsaw, Poland; anna.waszczuk-gajda@wum.edu.pl (A.W.-G.); wieslaw.jedrzejczak@wum.edu.pl (W.W.-J.); grzegorz.basak@wum.edu.pl (G.B.); 2 Department of Experimental Hematology, Medical University of Lodz, 93-510 Lodz, Poland; magdamalicka@gmail.com (M.W.); piotr.smolewski@umed.lodz.pl (P.S.); 3 Department of Mathematics and Information Science, Warsaw University of Technology, 00-662 Warsaw, Poland; e.sienkiewicz@mini.pw.edu.pl (E.S.); przemyslaw.biecek@gmail.com (P.B.); 4 Department of Hematology and Bone Marrow Transplantation, Medical University of Silesia, 40-032 Katowice, Poland; cauda.equina@wp.pl (A.K.); ghelbig@o2.pl (G.H.); 5 Department of Hematology, Institute of Hematology and Transfusion Medicine, 02-776 Warsaw, Poland; akolkowska@ihit.waw.pl (A.K.-L.); jbarankiewicz@ihit.waw.pl (J.B.); emaranda@ihit.waw.pl (E.L.-M.); 6 Maria Sklodowska-Curie National Research Institute of Oncology, 31-115 Cracow, Poland; monika.dlugosz-danecka@lymphoma.edu.pl (M.D.-D.); wjurczak@gmail.com (W.J.); 7 Maria Sklodowska-Curie Institute-Cancer Center, Gliwice Branch, 44-102 Gliwice, Poland; sebastian.giebel@io.gliwice.pl

**Keywords:** Bing-Neel syndrome, Waldenström macroglobulinemia, lymphoplasmacytic lymphoma, central nervous system involvement, ibrutinib, BTK inhibitors

## Abstract

Bing-Neel syndrome (BNS) is a rare presentation of Waldenström macroglobulinemia (WM). BNS is a consequence of the central nervous system (CNS) involvement by lymphoplasmacytic lymphoma (LPL) and, rarely, the peripheral nervous system. The data on BNS are extremely scarce. Therefore, we performed a multicenter retrospective analysis of BNS patients diagnosed and treated in centers aligned with the Polish Lymphoma Research Group. The analysis covers the years 2014–2021. Eleven patients were included, 55% females and the median age at BNS diagnosis was 61 years. The median time from WM to BNS was 3.5 years; 27% of patients did have a diagnosis of WM and BNS made simultaneously or within 30 days from each other. Isolated parenchymal involvement was the least frequent (20%). Patients were treated with different regimens, mostly able to cross the blood-brain barrier, including 18% treated with ibrutinib first line. The cumulative objective response to treatment was 73%. With the median follow-up of 20 months (95% CI, 2–32), the 36-month estimates were: overall survival (OS) 47%, progression-free survival (PFS) 33%, and cumulative incidence of BNS-associated death 41%. The performance status according to ECOG was significant for PFS (HR = 7.79) and the hemoglobin concentration below 11 g/dL was correlated with PFS. To conclude, BNS is a very rare manifestation of WM. It is associated with a poor outcome with most patients succumbing to BNS.

## 1. Introduction

Bing-Neel syndrome (BNS), described for the first time by Jens Bing and Axel Valdemar von Neel in 1936, is a rare disease presentation of Waldenström macroglobulinemia (WM), which is self a very uncommon B-cell lymphoid malignancy [1,2]. In the study of Kulkarni et al., only 13 BNS cases were identified among 1523 patients diagnosed with WM between the years 1999 and 2013, allowing for the calculation of a relative frequency of BNS of <1% [3]. BNS is a consequence of the central nervous system involvement by lymphoplasmacytic lymphoma (LPL) and, rarely, the peripheral nervous system (PNS). The clinical presentation of BNS may be very diverse, with no pathognomonic symptoms, which together with the extremely rare occurrence of BNS makes the appropriate diagnosis challenging. Additionally, the similarity of some of the BNS symptoms to the symptoms observed in other complications of WM, e.g., hyperviscosity syndrome or neuropathy related to anti-myelin associated glycoprotein (MAG) antibodies [4], makes the diagnosis even more challenging for physicians. The difficulties may be further aggravated by the fact, that BNS may occur in any stage of WM, including patients with known WM, even in the absence of systemic progression, but also in previously undiagnosed patients.

Because of the rarity of the disease, the data on the characteristics of BNS patients, treatment of the disorder, as well as the prognosis are extremely scarce based mostly on case reports [5,6,7] and two more numerous groups described by Simon et al. [8] and Castillo et al. [9]—for details please see Table 1. Therefore, taking into consideration the scarcity of data on BNS and simultaneously the need for increasing the awareness of the physicians of the diagnosis of BNS we at the Polish Lymphoma Research Group conducted a study on BNS in Polish patients.

## 2. Materials and Methods

### 2.1. Data Source

The study was performed on behalf of the Indolent Lymphomas Working Party of the Polish Lymphoma Research Group (PLRG). PLRG is a voluntary organization comprising hematological and oncological centers in Poland providing care for lymphoma patients [12].

All member centers were invited to participate in this study and provide additional study-specific data about eligible patients.

### 2.2. Study Population and Outcome

This study was a retrospective analysis of all patients who were diagnosed with BNS either simultaneously or sequentially with WM. The analysis includes patients diagnosed with BNS in the period 2014–2021.

The primary objective of the study was to analyze the outcome of BNS, i.e., overall survival (OS) of the patients with BNS and mortality from BNS. The secondary objectives were to examine the clinical presentation of BNS, the efficacy of BNS treatment, progression-free survival (PFS), and factors associated with OS and PFS.

### 2.3. Diagnosis of BNS

According to published guidelines [4], a diagnosis of BNS was made in cases (1) when a biopsy of the cerebrum or meninges demonstrated an LPL, or (2) LPL cells were detected by flow cytometry in the cerebrospinal fluid (CSF) with evidence of the clonality of the cells. LPL was defined as a lymphoma composed of small B lymphocytes, plasmacytoid lymphocytes, and plasma cells with immunophenotype: sIgM+, cIg, CD19+, CD20+, CD22+, CD79a+, CD5−, CD10−, CD103−, CD23−/+, CD138+ (plasma cells), CD45+/−, CD25−/+. Detection of *MYD88* L265P mutation in CSF was not sufficient for the diagnosis of proven BNS.

In cases without histopathological confirmation of BNS in patients previously diagnosed with WM, but with radiological and clinical symptoms suggestive of BNS and abnormal CSF tests (elevated cell count with presence of pathological cells, increased protein concentration, positive immunofixation for immunoglobulin M) a diagnosis of clinically diagnosed BNS was allowed for this analysis. 

### 2.4. Response to Treatment

Response to treatment was assessed as proposed by a task force on BNS established during the 8th International Workshop on WM [4] with modification for uncertain responses as in the studies of Castillo et al. [9] and Simon et al. [8]. The response categories comprised: (1) complete remission (CR), defined as resolution of all reversible clinical symptoms with normalization of CSF and magnetic resonance imaging (MRI) findings (MRI findings could show minimal residual abnormalities on T2 or FLAIR); (2) partial response (PR) defined as an improvement but no complete resolution of all reversible clinical symptoms, or complete resolution of all reversible clinical symptoms but with maintained radiological abnormalities, excluding minimal residual abnormalities on T2 or FLAIR; negative CSF; (3) non-response (NR) defined as persistence or progression of neurological symptoms, radiological or CSF findings; (4) relapse defined as the reappearance of new signs and symptoms attributed to BNS; or detection by cytological, and/or flow cytometry, and/or molecular techniques of BNS disease; or progression or new findings attributed to BNS by MRI examination of the brain and/or spine. For patients lacking complete data on all tests necessary for response assessment, a category of uncertain complete remission (uCR) was applied. uCR was defined as resolution of all reversible clinical symptoms with either normalization of MRI findings but without CSF evaluation or normalization of CSF but without MRI evaluation.

Response of BNS was assessed independently of response of WM on bone marrow or protein level.

### 2.5. Statistical Analysis

All time-to-event outcomes were computed from the day of BNS diagnosis. OS was defined as the time from the day of BNS diagnosis to death from any cause, while PFS was the time from BNS diagnosis to death or relapse, whichever occurred first. The cumulative incidence (CI) of death due to BNS was defined as the time from the day of BNS diagnosis to death, with death from other than BNS causes considered a competing event.

The Kaplan–Meier estimator and log-rank test were used for OS, and the crude cumulative incidence estimator and Gray’s test were used for competing events (death from BNS vs. death from other causes). The median follow-up was calculated using the reverse Kaplan–Meier estimator.

The Cox proportional hazards regression model was used in univariate analysis for comparisons of groups, with a cause-specific model for the outcomes of competing risks. Pearson’s chi-squared test was used to test the independence of categorical variables.

*p*-values < 0.05 were considered significant. All estimates are reported with accompanying 95% confidence intervals in brackets. Continuous variables are presented as median values (and their range), while frequency tables are used for categorical variables. All analyses were performed using the statistical software R version 4.0.2.

## 3. Results

### 3.1. Patients

Eleven patients diagnosed with BNS were identified among 201 patients with WM staying under charge in five centers. Other centers declared they did not diagnose and treat more patients with BNS. Six (55%) patients were females, and the median age at BNS diagnosis was 61 years (range, 47–72). The median time from WM to BNS diagnosis was 3.5 years (range, 0–17.2), with 3 (27%) patients having either a simultaneous diagnosis or a diagnosis of BNS made within 30 days from WM diagnosis. Most patients were heavily pretreated for WM with the median number of previous lines of therapy of 3 (range, 0–7). For details, please see Table 2.

The clinical symptoms were diverse, with headaches (36%), gait disorders (36%), paresis (27%), and sensory symptoms (27%) being the most frequent (Table 2). The median time from the symptoms’ onset to BNS diagnosis was 2.3 months (range, 0.5–63.9), with only one patient (10%) having a diagnosis made after more than a year.

### 3.2. Diagnosis

The diagnostic procedures are summarized in Table 3. Imaging was performed only in 9 (82%) patients, with 8 (73%) having performed an MRI. No patient had a biopsy of a CNS lesion. All patients had a CSF examination, with abnormal results in all of them. Flow cytometry (FC) allowed for the identification of LPL cells in 7 out of 7 examined patients, which resulted in a diagnosis of proven BNS. The other 4 patients did have a clinically diagnosed BNS, with all of them having pathological cells detectable in the CSF. Among patients with clinically diagnosed BNS, three patients did have an elevated cell count along with an increased protein concentration. Two of these patients had positive immunofixation for IgM in the CSF, and one positive genetic testing for *MYD88* L265P. The exact information on the cell count and protein concentration in the CSF was missing for one patient. In general, protein electrophoresis and immunofixation were performed only in a minority of patients, similarly, genetic studies aimed at the detection of *MYD88* L265P.

Based on the performed studies, meningeal involvement was found in 8 (80%), and parenchymal involvement in 6 (60%), including two (20%) patients with isolated parenchymal involvement among 10 patients, for whom the data was available.

### 3.3. Treatment

All patients received treatment for BNS. A variety of different protocols was used in the first line, most frequently high doses of methotrexate and/or cytarabine or DRC (dexamethasone, rituximab, cyclophosphamide). Two patients (18%) were treated with ibrutinib, one at a dose of 420 mg, and one at 560 mg. Two patients (18%) required salvage treatment because of insufficient response. One patient underwent autologous hematopoietic cell transplantation (auto-HCT) for consolidation after the second-line treatment, after BCNU/etoposide/thiotepa conditioning, as described in [13]. One patient required third-line treatment, ibrutinib. No patient underwent allogeneic hematopoietic cell transplantation. Intrathecal chemotherapy was administered to 7 (64%) patients, always along with systemic treatment. Plasmaphereses were performed in 3 patients (27%), in 2 (18%) for hyperviscosity syndrome, and in 1 (9%) for IgM neuropathy. For details, please see Table 3.

Among patients eligible for response assessment all obtained objective response after the completion of the first line and salvage treatment, with four patients (50%) obtaining either complete response or uncertain complete response; please see Table 3. The response in patients treated with ibrutinib was CR (1 patient) and PR (1 patient) in the first line, and uCR in the only patient treated with ibrutinib in the salvage setting.

Patients with sole parenchymal involvement were treated with either ibrutinib 560 mg daily or with DRC along with intrathecal therapy, with both obtaining solely PR.

### 3.4. Survival

**Figure 1:**
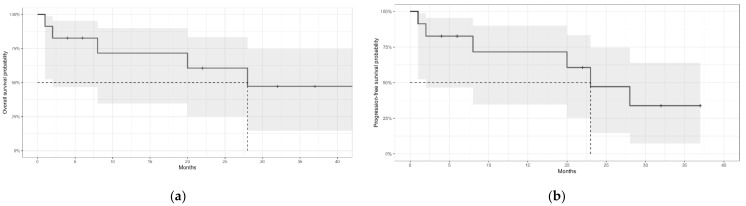
(**a**) Overall survival of patients with Bing-Neel syndrome. (**b**) Progression-free survival of patients with Bing-Neel syndrome. Grey areas represent the 95% confidence intervals. The dotted lines indicate the median survival.

With a median follow-up of 20 months (95% CI, 2–32), the median OS was 28 months (95% CI, 2-NA). The 12-month OS was 71% (95% CI, 34–90), the 24-month OS-60% (95% CI, 25–83), and the 36-month OS-47% (95% CI, 14–74). Please see Figure 1a. The median PFS was 23 months (95% CI, 2-NA), with a 12-month estimate of 71% (95% CI, 34–90), a 24-month estimate of 47% (95% CI, 14–74), and a 36-month estimate of 33% (95% CI, 7–64). Please see Figure 1b.

The cumulative incidence of BNS-associated death was 29% (95% CI, 20–39) at 12 months and 41% (95% CI, 31–51) at 24 and 36 months. The CI of non-BNS-associated death was 0% (95% CI, 0–0) at 12 and 24 months and 14% (95% CI, 5–23) at 36 months.

Among the parameters tested, i.e., sex, age at BNS diagnosis (≤61 vs. >61 years), performance status according to ECOG (0–2 vs. 3–4), the timing of BNS diagnosis (simultaneous vs. subsequent), time from the onset of the symptoms to BNS diagnosis (≤2 months vs. >2 months), presence of extramedullary disease (other than CNS), presence of neuropathy, IgM concentration (<10 g/L vs. ≥10 g/L), hemoglobin concentration (<11 g/dL vs. ≥11 g/dL), disease localization (meningeal only vs. other), only the performance status according to ECOG was significant for PFS (HR = 7.78, 95% CI: 4.30–14.01), with higher ECOG score associated with shorter PFS. Moreover, lower hemoglobin concentration was associated with shorter PFS, but its influence on the hazard function could not be estimated.

## 4. Discussion

The manuscript summarizes the multi-center experience with the diagnosis and treatment of Bing-Neel syndrome, a rare presentation of Waldenström macroglobulinemia. The size of the described cohort of patients reflects the rarity of both WM itself and BNS, which develops in fewer than 1% of all patients with WM [3]. It cannot be excluded, that in the centers participating in the current PLRG study some of the patients did succumb to BNS before a correct diagnosis had been made. The existence of such a phenomenon has been suggested for BNS patients by the task force for BNS [4].

Despite the fact, that histological biopsy of the cerebrum or meninges demonstrating an LPL is the golden standard for the diagnosis of BNS, no patient in our group did have such a biopsy performed. Additionally, even though all patients did have an abnormal result of the CSF sample, not all of them had a flow cytometry performed to confirm the clonality of LPL cells. The lack of compliance with the recommended diagnostic procedures could at least partially be explained by the fact, that the patients without FC analysis had been diagnosed mostly during the earliest years covered by the analysis before the consensus guidelines were published [4]. Electrophoresis of CSF was not performed in most patients, reflecting the lack of the possibility of performing the analysis in most centers.

In the analyzed group, BNS did develop in patients with known WM, but also in previously undiagnosed patients. Nearly one-third of patients did have a simultaneous diagnosis of WM and BNS or a diagnosis of BNS made within 30 days of WM diagnosis, which is in line with other reports [8,9]. The simultaneous diagnosis of WM and BNS was not associated with improved outcomes, unlike in the study of Castillo et al. [9]. The group was however small, and the data needs to be interpreted with caution.

The clinical presentation of BNS was diverse, similarly to other studies, e.g., [8,9] indicating either meningeal or brain parenchyma/spinal cord involvement. The patients complained mostly of headaches (36%), gait disorders (36%), paresis (27%), and sensory symptoms (27%), nevertheless also many other less frequently occurring symptoms were reported by the patients. A significant proportion of patients (60%) suffered from peripheral neuropathies, which could have obscured the symptoms of BNS and delayed the diagnosis. Adequately to the reported symptoms, the patients did have either diffuse (meningeal or both meningeal and parenchymal) or parenchymal involvement. The most prevalent was isolated meningeal involvement, while isolated parenchymal involvement was the least frequent. These observations are in line with the observations made by others; e.g., in the study of Simon et al., only 9% of patients did have a tumoral BNS, while others did have a diffuse form [8].

As it is well known, no treatment guidelines exist for BNS. Consensus recommendations for the treatment approach of BNS were proposed in 2017 by the task force for BNS, which for symptomatic patients with BNS suggests administration of purine analogs or bendamustine or ibrutinib or HD-MTX-based or HD-AraC-based protocols [4]. More recently Castillo and Treon suggested using ibrutinib in the frontline setting if available and not previously used for the treatment of systemic WM [14]. This lack of recommendations is reflected by the treatment administered in our group. The patients were treated with a variety of different protocols, mostly combining drugs able to pass the blood-brain barrier used typically for the treatment of primary central nervous system lymphoma. Two patients were treated with ibrutinib in the first line and one in the salvage setting. Alike methotrexate and cytarabine, ibrutinib is also known to pass the blood-brain barrier [15,16,17,18,19]. As was shown by Castillo et al. ibrutinib given at a daily dose of 420 or 560 mg daily is very effective in the treatment of BNS, with 85% of patients having improvement or resolution of BNS symptoms, 83% having improvement or resolution of radiologic abnormalities, and 47% having a clearance of the disease in the CSF at the best response [10]. Moreover, other Bruton’s tyrosine kinase inhibitors (BTKi) have been used for BNS in single cases, e.g., zanubrutinib [20] or tirabrutinib [21]. Hematopoietic cell transplantation was performed solely on one patient in the analyzed group as part of salvage treatment. The conditioning comprised BCNU, thiotepa, and etoposide, the protocol dedicated to CNS lymphomas [13]. The literature data on auto-HCT in BNS are extremely scarce. Simon et al. summarized the French experience with auto-HCT in BNS, covering solely 14 patients transplanted between 1999–2018 [11]. In their analysis, auto-HCT was used as part of the first-line strategy in 11 patients, and salvage treatment—in the further 3 patients. Despite the limitations of the study, the authors were able to conclude, that auto-HCT was a good option for patients with chemosensitive diseases.

It is important to note that BNS like WM and other indolent lymphomas is incurable, therefore, the treatment aims to reverse the clinical symptoms and induce long-term PFS [4]. Unfortunately, in the analyzed group both PFS, with a 36-month estimate of 33%, and OS, with a 36-month estimate of 47%, were very unsatisfactory. These outcome results are worse than the results by Castillo et al. [9] and Simon et al. [8] who reported a 3-year OS of 59% and a 5-year OS of 71%, respectively. In our group, similarly to the study of Simon et al. [8], most patients succumbed to BNS and not to other comorbid conditions. Therefore, it seems reasonable to search for more efficacious therapeutics as well as to optimize the available treatment. An interesting option could be sequential monitoring of *MYD88* L265P burden in the CSF to aid therapeutic decisions including the information on the required intensity of treatment as suggested by Frustaci et al. [22].

The prognostic factors for the outcome identified in univariate analysis were the performance status according to ECOG and hemoglobin concentration, both significant for PFS. It is worth noting, that in the study of Castillo et al. [9], also age above 65 years, previous treatment for WM, and platelet count <100 × 10^9^/L were found to be adverse prognostic factors.

The limitations of the study are its retrospective nature and a very small sample size. The rarity of the diagnosis precludes however the possibility of gathering a large group of patients, even if performed muti-nationally. Nevertheless, we believe the study provides important information on BNS. It also indicates that appropriate handling of BNS patients is challenging and points to the fact, that appropriate education to increase physician awareness of the existence of this syndrome is necessary to aid the proper diagnostic workup and treatment of the patients.

## 5. Conclusions

Bing-Neel syndrome is an extremely rare presentation of Waldenström macroglobulinemia. It is associated with dismal outcomes. The cause of death in most patients is associated with BNS. Obtaining a response to treatment is possible in most patients. Ibrutinib administration is effective both in the first and in the later lines of treatment.

## Figures and Tables

**Table 1:** Patients’ characteristics (auto-HCT—autologous hematopoietic cell transplantation; BDR—bortezomib, dexamethasone, rituximab; BNS—Bing-Neel syndrome; BR—bendamustine, rituximab; CNS—central nervous system; DRC—dexamethasone, rituximab, cyclophosphamide; ECOG—Eastern Cooperative Oncology Group; FLC—free light chains; IgG—immunoglobulin G; IgM—immunoglobulin M; IPSSWM—International Prognostic Scoring System for Waldenström macroglobulinemia; OR—objective response; SD—stable disease; PD—progressive disease; WM—Waldenström macroglobulinemia).

	Number of Patients	Median Age at BNS (Range)	Sex (Males)	Median Time from WM to BNS (Range)	Simultaneous Diagnosis	The Most Frequent Symptoms	Localization	First-Line Treatment	ORR to the First-Line Treatment	Overall Survival	Progression-Free Survival	Adverse Prognostic Factors
Castillo et al. [9]	34	62 (39–76)	19 (56%)	3 years (0–16)	15%	Motor deficits of the limbs (35%); altered mental status (35%), cranial nerve symptoms (29%)	NR	HD-MTX-based (41%); Intrathecal-based (19%); HD-MTX + HD-AraC-based (16%)	66%	3-year: 59% (95% CI, 39–75%)	NR	Age > 65 years; PLT < 100 × 10^9^/L; treatment for WM prior to BNS
Simon et al. [8]	44	63 (47–84 )	35 (80%)	8.9 years (0.8–25) (non-simultaneous diagnosis)	36%	Balance disorder/ disturbed gait (48%); cranial nerve symptoms (36%); cognitive impairment (27%)	Diffuse-93%; Tumoral-9%	HD-MTX-based (41%); Intrathecal-based (9%); HD-MTX + HD-AraC-based (9%)	70%	5-year: 71%; 10-year 59%	Median 26 months	Not analyzed
Kulkarni et al. [3]	13	60 (51–75)	NR	6.3 years (0.3–11.9)	0%	Seizures, hearing loss, cognitive impairment, gait instability, lower extremity weakness	NR	HD-MTX-based (62%); Intrathecal-based (23%); BR (8%)	45%	Median not reached	NR	Not analyzed
Varettoni et al. [5]	4	62 (38–68)	3 (75%)	NR	75%	Motor deficits (50%); ataxia (50%); cognitive impairment (25%); seizures (25%)	Diffuse-75%; Tumoral-25%	Intrathecal-based (75%); BR (75%)	75%	NR	NR	Not analyzed
Patients treated with a specific therapy												
Vos et al. [6]—only patients treated with fludarabine	4	60 (41–70)	1 (25%)	simultaneous	100%	Motor deficits (50%); cognitive impairment (50%); sensory deficits (25%); seizures (25%)	Diffuse-100%	Fludarabine-based (100%)	100%	2-year OS 100%	2-year PFS 100%	Not analyzed
Castillo et al. [10]- only patients treated with ibrutinib	28	65 (38–81)	16 (57%)	4.0 years (0–26.7)	NR	Motor deficits (46%); cognitive impairment (39%); sensory deficits (39%)	NR	Ibrutinib	41%	2-year Ibrutinib-OS 81% (95% CI, 49–94%)	2-year EFS 80%	Not identified
Simon et al. [11]– only patients treated with auto-HCT	14	61 (52–67)	9 (64%)	NR	NR	NR	NR	HD-AraC-based (57%); HD-MTX-based (7%); HD-MTX + HD-AraC-based (7%)	NR	NR	NR	Not analyzed

**Table 2:** Patients’ characteristics (auto-HCT—autologous hematopoietic cell transplantation; BDR—bortezomib, dexamethasone, rituximab; BNS—Bing-Neel syndrome; BR—bendamustine, rituximab; CNS—central nervous system; DRC—dexamethasone, rituximab, cyclophosphamide; ECOG—Eastern Cooperative Oncology Group; FLC—free light chains; IgG—immunoglobulin G; IgM—immunoglobulin M; IPSSWM—International Prognostic Scoring System for Waldenström macroglobulinemia; OR—objective response; SD—stable disease; PD—progressive disease; WM—Waldenström macroglobulinemia).

	N (%)
Number of patients	11
Sex	
Female	6 (55%)
Male	5 (45%)
**At WM diagnosis**	
Age at WM diagnosis, median (range), years	59 (32–65)
Previous MGUS	1 (9%)
Performance status according to ECOG (missing: 2)	
0	2 (22%)
1	3 (33%)
2	2 (22%)
3	1 (11%)
4	1 (11%)
IPSSWM (missing: 2)	
Low	3 (33%)
Intermediate	4 (44%)
High	2 (22%)
**At BNS diagnosis**	
Age at BNS diagnosis, median (range), years	61 (47–72)
Performance status according to ECOG (missing: 1)	
0	0 (0%)
1	3 (30%)
2	2 (20%)
3	3 (30%)
4	2 (20%)
Time from WM diagnosis to BNS, median (range), years	3.5 (0–17.2)
Simultaneous WM and BNS diagnosis	1 (9%)
Diagnosis of BNS within 30 days from WM diagnosis	2 (18%)
Number of lines of therapy for WM before BNS diagnosis, median (range)	3 (0–7)
Previous treatment (any line)	
Chlorambucil	2 (18%)
Fludarabine/cladribine	5 (45%)
BR	1 (9%)
DRC	4 (36%)
BDR	2 (18%)
Rituximab (missing: 1)	5 (50%)
Auto-HCT for WM before BNS diagnosis	1 (9%)
Status of WM at BNS diagnosis (missing: 1)	
OR	1 (10%)
SD	1 (10%)
PD	3 (30%)
On treatment before response assessment	3 (30%)
Untreated	2 (20%)
Extramedullary WM disease (other than CNS), (missing: 1)	4 (40%)
Peripheral neuropathy, (missing 1)	6 (60%)
Laboratory parameters	
Hemoglobin, median (range), g/dL, (missing: 1)	10.3 (8.3–14.8)
Platelets, median (range), ×10^9^/L, (missing: 1)	190 (15–288)
Neutrophils, median (range), ×10^9^/L, (missing: 1)	2.5 (0.4–4.9)
Lymphocytes, median (range), ×10^9^/L, (missing: 1)	1 (0.1–1.5)
IgM concentration, median (range), g/L, (missing: 1)	10.2 (1.9–95.7)
IgG concentration, median (range), g/L, (missing: 2)	7 (1.4–13)
FLC kappa/lambda ratio (missing: 1)	2.01 (0.004–691)

**Table 3:** Disease characteristics (allo-HCT—allogeneic hematopoietic cell transplantation; AraC—cytarabine; auto-HCT—autologous hematopoietic cell transplantation; BNS—Bing-Neel syndrome; BR—bendamustine, rituximab; CNS—central nervous system; CR—complete remission; CSF—cerebrospinal fluid; CT—computed tomography; DRC—dexamethasone, rituximab, cyclophosphamide; HD—high dose; IgM—immunoglobulin M; MRI—magnetic resonance imaging; MTX—methotrexate; NR—non-response; PR—partial remission; uCR—uncertain complete remission; VCD—bortezomib, cyclophosphamide, dexamethasone).

	N (%)
Number of patients	11
Time from the symptoms onset to diagnosis, median (range), months (missing: 1)	2.3 (0.5–63.9)
Symptoms	
Headaches	4 (36%)
Gait disorders	4 (36%)
Sensory symptoms	3 (27%)
Cognitive deficits	2(18%)
Dysarthria	1 (9%)
Confusion	1 (9%)
Depressed level of consciousness	0 (0%)
Paresis	3 (27%)
Seizures	1 (9%)
Cranial nerve involvement (facial nerve)	1 (9%)
Visual disturbances	2 (18%)
Hearing impairment/hearing loss	2 (18%)
Psychiatric symptoms	1 (9%)
**Diagnostic procedures**	
MRI of the brain and spinal cord	8 (73%)
Abnormal	7/8 (88%)
CT of the brain	1 (9%)
Cerebrospinal fluid	
Leukocytes in CSF, median (range), /mm^3^ (missing: 2)	33 (3–214)
Protein in CSF, median (range), mg/dL (missing: 1)	188 (32–1616)
Flow cytometry analysis of CSF (missing: 1)	7 (70%)
Abnormal result	7/7 (100%)
Protein electrophoresis of CSF	2 (18%)
Abnormal	1/2 (50%)
Immunofixation of CSF	4 (36%)
IgM	4/4 (100%)
Analysis of *MYD88* L265P in CSF (missing: 1)	3 (30%)
Detectable *MYD88* L265P in CSF	3/3 (100%)
Biopsy of a CNS lesion	0 (0%)
**Disease localization**	
Disease localization (missing: 1)	
Meningeal involvement	4 (40%)
Parenchymal involvement	2 (20%)
Both meningeal and parenchymal	4 (40%)
Encephalon involvement (missing: 1)	5 (50%)
Spinal cord involvement (missing: 1)	1 (10%)
**Treatment of BNS**	
The first line systemic therapy for BNS (N = 11)	
HD-MTX-based	4 (36%)
HD-AraC-based	2 (18%)
DRC	3 (27%)
BR	1 (9%)
VCD	1 (9%)
Ibrutinib	2 (18%)
420 mg	1 (9%)
560 mg	1 (9%)
Rituximab (in combination)	6 (55%)
Radiotherapy	1 (9%)
The 1st salvage systemic therapy for BNS (N = 2)	
HD-MTX/HD-AraC	1 (50%)
HD-AraC-Temozolomid + auto-HCT	1 (50%)
The 2nd salvage systemic therapy for BNS (N = 1)	
Ibrutinib (420 mg)	1 (100%)
Intrathecal therapy	7 (64%)
Plasmaphereses	3 (27%)
Allo-HCT for BNS	0 (0%)
Cumulative response to treatment	
CR	2 (18%)
uCR	2 (18%)
PR	4 (36%)
NR	0 (0%)
Early death during treatment	1 (9%)
Not assessed	2 (18%)

## Data Availability

The data are available from the corresponding author.

## References

[1] Kastritis E., Leblond V., Dimopoulos M.A., Kimby E., Staber P., Kersten M.J., Tedeschi A., Buske C., ESMO Guidelines Committee (2018). Waldenström’s macroglobulinaemia: ESMO Clinical Practice Guidelines for diagnosis, treatment and follow-up. Ann. Oncol..

[2] Wang H., Chen Y., Li F., Delasalle K., Wang J., Alexanian R., Kwak L., Rustveld L., Du X.L., Wang M. (2012). Temporal and geographic variations of Waldenstrom macroglobulinemia incidence: A large population-based study. Cancer.

[3] Kulkarni M.T., Treon S.P., Manning R., Xu L., Rinne M., Lee E.Q., Ghobrial I.M., Norden A., Kluk M.J., Nayak L. (2013). Clinical Characteristics and Treatment Outcome of CNS Involvement (Bing-Neel Syndrome) in Waldenstrom’s Macroglobulinemia. Blood.

[4] Minnema M.C., Kimby E., D’Sa S., Fornecker L.M., Poulain S., Snijders T.J. (2017). Guideline for the diagnosis, treatment and response criteria for Bing-Neel syndrome. Haematologica.

[5] Varettoni M., Defrancesco I., Diamanti L., Marchioni E., Farina L.M., Pichiecchio A. (2017). Bing-Neel Syndrome: Illustrative Cases and Comprehensive Review of the Literature. Mediterr. J. Hematol. Infect. Dis..

[6] Vos J.M.I., Kersten M.-J., Kraan W., Groeneveld O.N., Linn C., Pals S.T., Minnema M.C. (2016). Effective treatment of Bing-Neel Syndrome with oral fludarabine: A case series of four consecutive patients. Br. J. Haematol..

[7] Varettoni M., Marchioni E., Bonfichi M., Picchiecchio A., Arcaini L., Arbasino C., Gotti M., Da Vià K., Delmonte M., Sciarra R. (2015). Successful treatment with Rituximab and Bendamustine in a patient with newly diagnosed Waldenström’s Macroglobulinemia complicated by Bing-Neel syndrome. Am. J. Hematol..

[8] Simon L., Fitsiori A., Lemal R., Dupuis J., Carpentier B., Boudin L., Corby A., Aurran-Schleinitz T., Gastaud L., Talbot A. (2015). Bing-Neel syndrome, a rare complication of Waldenström macroglobulinemia: Analysis of 44 cases and review of the literature. A study on behalf of the French Innovative Leukemia Organization (FILO). Haematologica.

[9] Castillo J.J., D’Sa S., Lunn M., Minnema M.C., Tedeschi A., Lansigan F., Palomba M.L., Varettoni M., Garcia-Sanz R., Nayak L. (2016). Central nervous system involvement by Waldenström macroglobulinaemia (Bing-Neel syndrome): A multi-institutional retrospective study. Br. J. Haematol..

[10] Castillo J.J., Itchaki G., Paludo J., Varettoni M., Buske C., Eyre T.A., Chavez J.C., Shain K.H., Issa S., Palomba M.S. (2019). Ibrutinib for the treatment of Bing-Neel syndrome: A multicenter study. Blood.

[11] Simon L., Lemal R., Fornecker L.M., Tournilhac O., Leblond V. (2019). High-dose therapy with autologous stem cells transplantation in Bing-Neel syndrome: A retrospective analysis of 14 cases. Am. J. Hematol..

[12] Giebel S., Basak G., Bieniaszewska M., Czerw T., Czyż A., Drozd-Sokołowska J., Dytfeld D., Giannopoulos K., Gil L., Helbig G. (2021). Current status and achievements of Polish haemato-oncology. Acta Haematol. Pol..

[13] Korfel A., Elter T., Thiel E., Hänel M., Möhle R., Schroers R., Reiser M., Dreyling M., Eucker J., Scholz C. (2013). Phase II study of central nervous system (CNS)-directed chemotherapy including high-dose chemotherapy with autologous stem cell transplantation for CNS relapse of aggressive lymphomas. Haematologica.

[14] Castillo J.J., Treon S.P. (2019). How we manage Bing-Neel syndrome. Br. J. Haematol..

[15] Cabannes-Hamy A., Lemal R., Goldwirt L., Poulain S., Amorim S., Pérignon R., Berger J., Brice P., De Kerviler E., Bay J.-O. (2016). Efficacy of ibrutinib in the treatment of Bing-Neel syndrome. Am. J. Hematol..

[16] Bernard S., Goldwirt L., Amorim S., Brice P., Brière J., de Kerviler E., Mourah S., Sauvageon H., Thieblemont C. (2015). Activity of ibrutinib in mantle cell lymphoma patients with central nervous system relapse. Blood.

[17] Mason C., Savona S., Rini J.N., Castillo J.J., Xu L., Hunter Z.R., Treon S.P., Allen S.L. (2017). Ibrutinib penetrates the blood brain barrier and shows efficacy in the therapy of Bing Neel syndrome. Br. J. Haematol..

[18] Hartsell L., Janes A., Larck C., Park S., Arnall J.R. (2019). Ibrutinib for the treatment of Bing-Neel syndrome, a complication of Waldenström macroglobulinemia: Patient case report. J. Oncol. Pharm. Pract..

[19] Boudin L., Patient M., Roméo E., Bladé J.S., de Jauréguiberry J.P. (2018). Efficacy of ibrutinib as first-line treatment of tumoral Bing-Neel syndrome. Leuk. Lymphoma.

[20] Wong J., Cher L., Griffiths J., Cohen A., Huang J., Wang L., Gregory G., Opat S. (2018). Efficacy of Zanubrutinib in the Treatment of Bing-Neel Syndrome. Hemasphere.

[21] Oyama T., Taoka K., Chiba A., Matsuda K., Maki H., Masamoto Y., Kurokawa M. (2022). A Case of Bing-Neel Syndrome Successfully Treated with Tirabrutinib. Intern. Med..

[22] Frustaci A.M., Rusconi C., Picardi P., Veronese S., Montillo M., Cairoli R., Tedeschi A. (2016). Bing Neel Syndrome in a Previously Untreated Patient with Waldenström’s Macroglobulinemia: Contribution of MYD88 L265P Mutation on Cerebrospinal Fluid. Clin. Lymphoma Myeloma Leuk..

